# Pharmacokinetic profile of amodiaquine and its active metabolite desethylamodiaquine in Ghanaian patients with uncomplicated falciparum malaria

**DOI:** 10.1186/s12936-020-03553-6

**Published:** 2021-01-06

**Authors:** Thomas A. Anyorigiya, Sandra Castel, Katya Mauff, Frank Atuguba, Bernhards Ogutu, Abraham Oduro, David Dosoo, Kwaku-Poku Asante, Seth Owusu-Agyei, Alexander Dodoo, Abraham Hodgson, Fred Binka, Lesley J. Workman, Elizabeth N. Allen, Paolo Denti, Lubbe Wiesner, Karen I. Barnes

**Affiliations:** 1 Division of Clinical Pharmacology, Department of Medicine, University of Cape Town, Cape Town, South Africa; 2 UCT/MRC Collaborating Centre for Optimising Antimalarial Therapy (CCOAT), University of Cape Town, Cape Town, South Africa; 3 Navrongo Health Research Centre, Navrongo, Ghana; 4 Department of Statistical Sciences, University of Cape Town, Cape Town, South Africa; 5 Dodowa Health Research Centre, Dodowa, Ghana; 6 Centre for Clinical Research, Kenya Medical Research Institute, Nairobi, Kenya; 7 Kintampo Health Research Centre, Kintampo, Ghana; 8 University for Health and Allied Sciences, Ho, Volta Region Ghana; 9 Ghana Standards Authority, Accra, Ghana; 10 Research and Development Division, Ghana Health Service, Accra, Ghana

**Keywords:** Amodiaquine, Artesunate, Fixed-dose combination, Pharmacokinetics, *P. falciparum* malaria, Ghana, Infants, Young children, Underweight-for-age, Parasite density

## Abstract

**Background:**

Accurate measurement of anti-malarial drug concentrations in therapeutic efficacy studies is essential to distinguish between inadequate drug exposure and anti-malarial drug resistance, and to inform optimal anti-malarial dosing in key target population groups.

**Methods:**

A sensitive and selective LC–MS/MS method was developed and validated for the simultaneous determination of amodiaquine and its active metabolite, desethylamodiaquine, and used to describe their pharmacokinetic parameters in Ghanaian patients with uncomplicated falciparum malaria treated with the fixed-dose combination, artesunate-amodiaquine.

**Results:**

The day-28 genotype-adjusted adequate clinical and parasitological response rate in 308 patients studied was > 97% by both intention-to-treat and per-protocol analysis. After excluding 64 patients with quantifiable amodiaquine concentrations pre-treatment and 17 with too few quantifiable concentrations, the pharmacokinetic analysis included 227 patients (9 infants, 127 aged 1–4 years, 91 aged ≥ 5 years). Increased median day-3 amodiaquine concentrations were associated with a lower risk of treatment failure [HR 0.87 (95% CI 0.78–0.98), p = 0.021]. Amodiaquine exposure (median AUC_0-∞_) was significantly higher in infants (4201 ng h/mL) and children aged 1–5 years (1994 ng h/mL) compared to older children and adults (875 ng h/mL, p = 0.001), even though infants received a lower mg/kg amodiaquine dose (median 25.3 *versus* 33.8 mg/kg in older patients). Desethylamodiaquine AUC_0-∞_ was not significantly associated with age. No significant safety concerns were identified.

**Conclusions:**

Efficacy of artesunate-amodiaquine at currently recommended dosage regimens was high across all age groups. Reassuringly, amodiaquine and desethylamodiaquine exposure was not reduced in underweight-for-age young children or those with high parasitaemia, two of the most vulnerable target populations. A larger pharmacokinetic study with close monitoring of safety, including full blood counts and liver function tests, is needed to confirm the higher amodiaquine exposure in infants, understand any safety implications and assess whether dose optimization in this vulnerable, understudied population is needed.

## Background

Since 2000, there have been dramatic increases in political and financial commitments towards efforts to control malaria, with widespread deployment of artemisinin-based combination therapy (ACT) as first-line treatment of uncomplicated *Plasmodium falciparum* malaria, which has contributed to significant decreases in the malaria burden [1, 2]. Artesunate plus amodiaquine (ASAQ) is one of five artemisinin-based combinations currently recommended by the World Health Organization (WHO) [3] and was introduced as first-line treatment in Ghana in 2005 [4]. At that time, the therapeutic efficacy of ASAQ was 100% in children under five followed up for 28 days [5]. Subsequently, cure rates ranging from 80 to 100% were found in Ghanaian children aged 6 months to 10 years, the age group with the highest malaria burden [6–10].

Effective malaria treatment requires that the frequency and dose of anti-malarial drugs is adequate to provide sufficient drug concentrations over time to kill all parasites in all key target populations [11]. Sub-therapeutic drug exposures contribute to poorer treatment responses and may fuel the spread of anti-malarial resistance [12]. In therapeutic efficacy studies, inadequate drug exposure may be misinterpreted as parasite resistance unless drug concentrations are accurately measured [3]. Despite extensive use of amodiaquine for many years, previously as a monotherapy and currently in combination with artesunate for the treatment of uncomplicated falciparum malaria [13, 14] and in combination with sulfadoxine-pyrimethamine for Seasonal Malaria Chemoprevention [15, 16], pharmacokinetic data are limited particularly for the parent compound amodiaquine and in key target populations such as very young and malnourished children and patients with uncomplicated hyperparasitaemia [17–23].

Drug concentrations are usually measured in plasma, but collection of plasma samples is less suitable for field studies [24]. As amodiaquine and desethylamodiaquine accumulate in white blood cells [24–26] and it is the red blood cells that are parasitized, concentrations of amodiaquine and desethylamodiaquine in whole blood may reflect the concentrations acting on the malaria parasites more accurately than plasma concentrations. Previously described methods for the simultaneous determination of amodiaquine and desethylamodiaquine concentrations required relatively large sample volumes (0.1–1 mL) that are not ideal for repeated sampling in young children [26–29].

This study aimed to characterize the pharmacokinetic and pharmacodynamic (PK/PD) profile of amodiaquine when given with artesunate as a fixed-dose combination to treat uncomplicated *P. falciparum* malaria in non-pregnant patients of all ages, including infants, malnourished young children and those with a high parasitaemia. This required the development and validation of a whole blood assay using small blood volumes, in order to ensure its feasibility for use in routine therapeutic efficacy studies.

## Methods

### Study design and participants

The study was conducted in Kassena-Nankana and Kintampo districts in Ghana, as a pharmacokinetic sub-study of the INDEPTH Effectiveness and Safety Studies (INESS) of the therapeutic efficacy of fixed-dose artesunate-amodiaquine for the treatment of uncomplicated falciparum malaria. The Kassena-Nankana district is in northern Ghana, while the Kintampo district is in central Ghana, with a distance of about 400 km between the study sites.

Non-pregnant patients aged > 2 months, weighing > 4.5 kg with *P. falciparum* mono-infection of 1000–200,000 parasites/µL and fever (axillary temperature ≥ 37.5 °C or history of fever within 24 h), willing to comply with the study procedures and with written informed consent or consent/assent, were included in the study at both Kassena-Nankana (Navrongo) and Kintampo sites between August 2011 to February 2012. To achieve the required sample size data collection continued in the Navrongo site only from July 2012 to January 2013. Those who had taken medication within the previous two weeks with known anti-malarial effects, potential interactions with amodiaquine/desethylamodiaquine pharmacokinetics or any artesunate-amodiaquine contra-indications were excluded.

### Study procedures

Patients were given a fixed-dose combination of artesunate-amodiaquine (Coarsucam^**®**^/ASAQ Winthrop; Sanofi-Aventis, Maphar Laboratories, Morocco) with water based on WHO recommended dosage regimens by body weight to achieve an artesunate target of 4 mg/kg body weight (range 2 to 10 mg/kg) and amodiaquine target of 10 mg/kg body weight of amodiaquine base (range 7.5 to 15 mg/kg) once daily for 3 days [3]. All patients were followed up at the Navrongo site according to the WHO protocol [30], with slight modifications in Kintampo where follow up was on days 2, 7, 14 and 28.

The exact time of each observed dose was recorded. Patients were observed for at least 30 min after treatment; if vomiting occurred within 30 min, the full dose was repeated. Patients who vomited more than once were excluded and referred for appropriate hospital management. Treatment efficacy outcomes were classified as per 2009 WHO recommended methods [30]. Parasite identification and density were assessed on thin and thick blood smears by at least two independent microscopists, assuming a WBC count of 8000/µL; at least 100 high power fields were read before declaring a slide negative [30]. *Plasmodium falciparum* deoxyribonucleic acid (DNA) was extracted from dried filter paper blood spots using a modified Chelex method, as described previously [31]. The three highly polymorphic *P. falciparum* antigens, MSP-1, MSP-2 and GLURP were used in the analysis to differentiate recrudescences from new infections [32]. To increase sensitivity, the PCR analysis of each gene involved 2 rounds of amplification with nested primers used in the second round. Primers and amplification conditions used were adapted from Ranford-Cartwright [33]. Parasite recrudescence was defined as the presence of identical PCR products in the samples from day 0 and the day of parasite recurrence (day X). Such recrudescences were further classified based on published criteria [32, 33].

An adverse event was defined as a new symptom or sign that developed post treatment or the exacerbation of a symptom or sign present at baseline. Treatment safety was assessed based on clinical signs and symptoms at each scheduled visit and on any unscheduled visit and by haemoglobin assessments on days 0, (2), 7, 14 and 28. Haemoglobin concentrations were determined in Navrongo on days 0, 7, 14 and 28 using Hemocue Hb201 + photometers^®^ (HemoCue AB, SE-262 23 Ängelholm, Sweden) and in Kintampo on days 0, 2 and 28 using ABX Micros 60–OT haematology analyzer (HORIBA Ltd, France). No additional laboratory safety tests were performed.

### Amodiaquine and desethylamodiaquine quantification

Pharmacokinetic samples were placed in a sample storage boxes and stored at minus 70 °C at each site until shipment on dry ice to the analytical laboratory. Blood concentrations of amodiaquine and desethylamodiaquine were recovered from 20 µL capillary whole blood samples collected into labeled lithium heparin tubes on all visits according to the same study SOP used at both sites based on WHO recommended methods [24, 30]. Pharmacokinetic blood samples were collected prior to artesunate-amodiaquine administration on Days 0, 1, and 2. Whole blood concentrations were measured within 6 months of sample collection using a liquid chromatography tandem mass spectrometry assay developed and validated [34] in the Division of Clinical Pharmacology at the University of Cape Town. The assay consisted of a liquid–liquid extraction, followed by high performance liquid chromatography with tandem mass spectrometry detection. The combined accuracy (%Nom) and precision (%CV) statistics of the quality controls of amodiaquine and desethylamodiaquine during validation were between 93.9% and 108.3%, and 3.2% and 5.8%, respectively. (See Additional file 1 for further assay method details).

### Statistical analysis

The sample size was calculated to compare the exposure of desethylamodiaquine in malaria patients who achieved adequate clinical and parasitological response and those who failed treatment. At an alpha value of 0.05, a power of 0.80 and a cure rate of 92.5%, a sample size per site of 119 was estimated. Assuming a 10% loss to follow up, a total of 300 patients were targeted to be enrolled from the two sites in order to allow also for the comparison of exposure between similar numbers of patients under 5 years with older children and adults.

Efficacy outcome analysis was carried out using both intention-to-treat (ITT) and per protocol (PP) methods [30]. In line with the burden of disease in areas of high malaria transmission [35], age was categorized into infants (< 1 year), young children (aged 1–4 years), and older children and adults (≥ 5 years), after testing for any statistically significant differences between older children and adults. Data with skewed distributions such as parasite densities were log transformed before being compared using the normal approximation, one-way analysis of variance (ANOVA) with Bonferroni correction for multiple comparisons. The weight-for-age z-scores were calculated according to the WHO growth reference for children ≤ 5 years [36]**.**

### Pharmacokinetic analysis

Pharmacokinetic parameters of amodiaquine and desethylamodiaquine were determined by a non-compartmental analysis using Stata v15 (StataCorp, Texas, USA). Patients who received treatment with any anti-malarials (including amodiaquine) within two weeks prior to enrolment, were excluded from the study. Therefore, even if prior anti-malarial treatment was not self-reported, patients with quantifiable amodiaquine concentrations prior to dosing were deemed to have had recent anti-malarial treatment and were excluded from analysis. Patients with pre-dose quantifiable desethylamodiaquine concentrations, but with corresponding amodiaquine concentrations below the limit of quantification were however included in the analysis as this was interpreted as amodiaquine treatment given > 2 weeks ago. Biologically implausible individual concentrations were also excluded from the analysis. A drug concentration was considered biologically implausible if, in the absorption phase or near the C_max_, it was ≥ 25% higher or lower than the 2 measured concentrations at adjacent sampling times or if, once in the terminal elimination phase, there was a ≥ 50% rise or fall relative to the two adjacent sampling times or the concentration increased after two successive lower concentrations.

The area under the concentration–time curve to infinity, AUC_0-∞_ was estimated using the trapezoidal rule with exponential fit. At least 3 data points were required for the estimation of AUC_0-∞_ and the terminal elimination rate constant (k_e_). The terminal elimination half-life was calculated as ln(2)/k_e_. The maximum observed amodiaquine and desethylamodiaquine concentration (C_max_) and the first time of their occurrence (T_max_) were obtained directly from the observed concentration–time data. The apparent clearance (CL/F) and volume of distribution (Vd/F) were calculated as Total Dose/AUC_0-∞_ and Apparent Clearance/k_e_, respectively, with F for bioavailability. For determining median amodiaquine and desethylamodiaquine concentrations at each time point, concentrations below the limit of quantification (BLQ) were set to half the lower limits of quantification i.e. 0.3905 ng/mL for amodiaquine and 1.955 ng/mL for desethylamodiaquine. All BLQ values were set to zero prior to first study drug dose and missing thereafter for the estimation of pharmacokinetic parameters.

A multivariate linear regression analysis of the log-transformed pharmacokinetic parameters was conducted to test for the independent relationships between pharmacokinetic parameters and pre-defined covariates: age (age category), gender, nutritional status (weight-for-age z-score if ≤ 5 years and an additional category for patients > 5 years), mg/kg total dose, presence or absence of fever at enrolment, parasite density (≥ 100,000 versus < 100,000 asexual parasites per microlitre), anaemia (Hb < 8.0 versus Hb ≥ 8.0 g/dL) and site of sample collection (Navrongo versus Kintampo Health Research Centre). Cox-proportional hazard ratios with Breslow correction for ties were used to explore the effects of the days 2, 3, 7, 14 and 28 concentrations of amodiaquine and desethylamodiaquine on treatment response.

## Results

### Trial profile

**Fig. 1:**
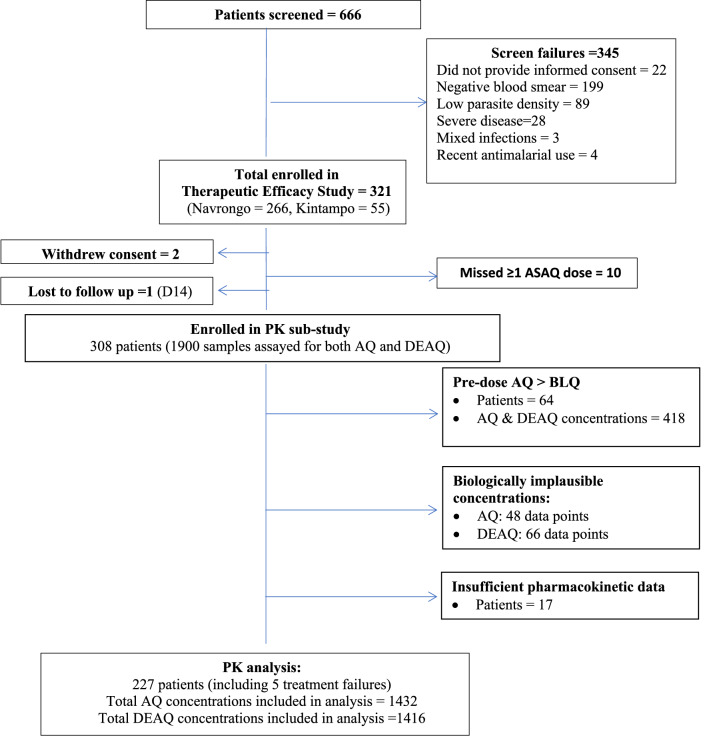
Trial profile of artesunate-amodiaquine fixed-dose combination treated Ghanaian patients with uncomplicated *P. falciparum* malaria

Of the 666 potential study participants screened, 321 patients were enrolled for the therapeutic efficacy trial, with 308 (96%) of these included in the pharmacokinetic study (Fig. 1). A total of 1900 samples were assayed for both amodiaquine and desethylamodiaquine concentrations. Of these, 64/308 (20.8%) had quantifiable amodiaquine concentrations prior to dosing, so were retrospectively excluded from the pharmacokinetic analysis. In addition, 48 amodiaquine and 66 desethylamodiaquine data points considered biologically implausible were excluded, resulting in a total of 1432 amodiaquine and 1416 desethylamodiaquine concentrations from 233 patients being included in the pharmacokinetic analysis; 17 patients had insufficient quantifiable drug concentrations for calculating pharmacokinetic parameters (Fig. 1).

### Baseline characteristics

Of the 308 patients enrolled into the pharmacokinetic study, 13 (4.2%) were infants (< 1 year), 176 (57.1%) children aged 1–4 years and 119 (38.6%) were aged 5 years or older (Table 1). Patients aged 5 years or older had lower baseline temperatures than younger patients (p = 0.017). Overall, 47/308 (15.3%) of the patients enrolled were moderate-to-severely anaemic (Hb < 8 g/dL), most of whom (37/47, 79%) were 1–4 years old. Of the children ≤ 5 years old, 14.7% (28/190) were underweight-for-age. The geometric mean parasite density at enrolment was 27,594 (95% CI 23,737–32,079)/µL overall and was lowest in infants (15,879 (95% CI 5,871–42,945)/µL compared to 34,921 (95% CI 28,916–42,172)/µL in children aged 1–4 years and 20,692 (95% CI 16,184–26,455)/µL in older patients (p = 0.001). The proportion of patients with parasite densities ≥ 100,000/µL was 15.3% (47/308), with most being children aged 1–4 years (32/47; 68.1%). Overall, the median total mg/kg dose administered was 33.8 (IQR 27.0–40.5) mg/kg, with the 25.8 mg/kg administered to infants lower than the 33.8 mg/kg given to patients aged 1–4 years (p = 0.004) or older (p = 0.010) (Table 1).

**Table 1:** Baseline characteristics of artesunate-amodiaquine fixed-dose combination treated Ghanaian patients with uncomplicated *P. falciparum* malaria

	Age category (years)			
	Total	< 1	1–4	≥ 5
N (%)	308 (100)	13 (4.2)	176 (57.1)	119 (38.6)
Sex, Female, n (%)	161 (52.3)	8 (61.5)	92 (52.3)	61 (51.3)
Underweight^b^ (WAZ < − 2.00), n/N (%)	28/190 (14.7)^a^	0 (0.0)	26 (13.6)	2
Axillary temperature (^o^C), mean (SD)	38.0 (1.1)	38.3 (0.8)	38.1 (1.1)	37.7 (1.2)
Haemoglobin (g/dL), mean (SD)	10.0 (1.9)	8.5 (1.3)	9.5 (1.8)	11.0 (1.8)
Moderate- to- severe anaemia patients (Hb < 8.0 g/dL), n (%)	47 (15.3)	5 (38.5)	37 (21.0)	5 (4.2)
Severe anaemia (Hb < 6.0 g/dL), n (%)	4 (1.3)	0 (0)	4 (2.3)	0(0)
Geometric mean parasite density, (95% CI)	27,594 (23,737–32,079)	15,879 (5871–42,945)	34,921 (28,916–42,172)	20,692 (16,184–26,455)
Parasite density ≥ 100,000, n (%)	47 (15.3)	3 (23.1)	32 (18.2)	12 (10.1)
Gametocyte Prevalence, n (%)	9 (2.9)	1 (7.7)	6 (3.4)	2 (1.7)
Total Dose (mg/ kg), median (IQR); range	31.2 (26.1–38.9) 7.6–52.6	25.3 (23.8–25.3) 13.4–31.15	33.8 (27.0–39.7) 14.5–45.0	33.8 (25.3–38.6) 7.2–52.6
D0 AQ concentration > LLOQ, n (%)	64 (20.8)	0(0)	45 (25.6)	19 (16.0)

N = Sample population, n = number of patients in category, WAZ = weight-for-age Z-score, sd = standard deviation, Hb = haemoglobin concentration, CI = confidence interval, mg/kg = milligram per kilogram, IQR = interquartile range, D0 = Day 0 pre-dose, AQ = amodiaquine, BLQ = below the limit of quantification

^a^ Two patients aged 5 years

^b^WAZ score only calculated for children ≤ 5 years; 2/13 (15.4%) children aged 5 years had WAZ score < − 2.00

### Efficacy

The crude (PCR–unadjusted) day 28 adequate clinical and parasitological response (ACPR) rate by intention-to-treat (ITT) analysis was 94.5% (291/308) [95% CI 91.3, 96.8] and by per protocol (PP) analysis was 95.2% (280/294) [95% CI 92.1, 97.4]. The overall day 28 PCR-adjusted ACPR rate was 98.0% (299/305) [95% CI 95.8, 99.3] by ITT and 98.0% (288/294 [95% CI 95.6–99.2] by PP analysis. There was no difference in efficacy by ITT between patients aged < 5 years (97.9%) and those aged 5 years or older (98.3%), p = 0.8 (Table 2). There were no early treatment failures. All recrudescences occurred on day 28 of follow up. Among patients who were re-dosed after vomiting post-dosing, 95.2% (20/21) achieved an ACPR with only 1 recrudescent infection. Parasite clearance was rapid, with only one patient (0.3%) still parasitaemic on day 3 post-treatment.

**Table 2:** Day 28 Therapeutic efficacy of artesunate-amodiaquine fixed dose combination treatment of uncomplicated *falciparum* malaria in Ghanaian patients, by Intention-to-treat (ITT) and Per Protocol (PP) analysis

Treatment outcome	Analysis	Age category (years)			
		Total	< 1	1–4	≥ 5
N	ITT	308	13	176	119
	PP	294	11	168	115
Early treatment failure (ETF), n (%)	ITT	0	0	0	0
	PP	0	0	0	0
Late clinical failure (LCF), n (%)	ITT	5 (1.6)	0	3 (1.7)	2 (1.7)
	PP	5 (1.7)	0	3 (1.8)	2 (1.7)
Late parasitological failure (LPF), n (%)	ITT	12 (3.9)	0	6 (3.5)	6 (5.0)
	PP	9 (3.1)	0	6 (3.6)	3 (2.6)
D28 PCR-uncorrected ACPR, (%)	ITT	*94.5*	*100*	*94.9*	*93.3*
	PP	*95.2*	*100*	*94.6*	*95.7*
Parasite recurrence, n	ITT	17	0	9	8
	PP	14	0	9	5
*P. falciparum* recrudescence on PCR, n	ITT	6	0	4	2
	PP	6	0	4	2
*P. falciparum* reinfection on PCR, n	ITT	8	0	5	3
	PP	8	0	5	3
^a^Indeterminate or missing PCR, n	ITT	3	0	0	3
	PP	0	0	0	0
Day 28 PCR-corrected ACPR, (%)	ITT	*98.0*	*100*	*97.7*	*98.3*
	PP	*98.0*	*100*	*97.6*	*98.3*

PCR: Polymerase chain reaction; ACPR: Adequate clinical and parasitological response

^a^Patients with indeterminate or missing PCR results were excluded from PP and ITT analyses

### Adverse events

A total of 136 adverse events were documented in 66 (21.4%) patients, many of which were consistent with features of malaria. All adverse events occurring within 28-days of follow up (Table 3) were mild or moderate and did not result in the discontinuation of treatment; there were no serious adverse events. The most common adverse events (occurring in > 1% of patients) were pyrexia (8.1%), cough (6.8%), asthenia (5.2%), abdominal pain (4.2%), upper respiratory tract infection (3.6%), headache (2.3%), diarrhoea (2.3%), decreased appetite (1.6%) and vomiting (1.3%) (Table 3). The number of patients with Adverse Events was not higher among infants despite their higher amodiaquine exposure than older patients (1/13, 8.3% versus 25/284, 8.1%; p = 0.98). Nor were the number of adverse events higher in infants than older patients (p = 0.93). The only infant who experienced adverse events developed gastroenteritis and an upper respiratory tract infection on day 42.

**Table 3:** Adverse events occurring within 28-days of  artesunate-amodiaquine fixed dose combination treatment of uncomplicated *falciparum* malaria, by Intention-to-treat analysis

Adverse event	Number of patients reporting event n (%)
Pyrexia	25 (8.1)
Cough	21 (6.8)
Asthenia	16 (5.2)
Abdominal pain	13 (4.2)
Upper respiratory tract infection	11 (3.6)
Headache	7 (2.3)
Diarrhoea	7 (2.3)
Decreased appetite	5 (1.6)
Vomiting	4 (1.3)
Gastroenteritis	3 (0.97)
Pruritis	3 (0.97)
Peripheral swelling	3 (0.97)
Pain	2 (0.65)
Oral pain	2 (0.65)
Restlessness	2 (0.65)
Otorrhoea	2 (0.65)
Dysentery	2 (0.65)
Furuncle	2 (0.65)
Abdominal distension	1 (0.32)
Dyspnea	1 (0.32)
Rash	1 (0.32)
Total	136 (44.2)

## Pharmacokinetic parameters of amodiaquine and desethylamodiaquine

### Amodiaquine

**Fig. 2:**
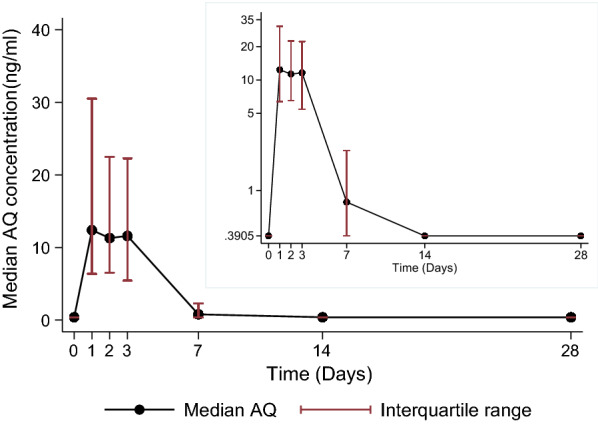
A plot of the median (IQR) capillary whole blood amodiaquine (AQ) concentrations versus time overlaid with log-transformed y-axis plot of median (interquartile range) capillary whole blood amodiaquine concentrations (ng/ml) versus time in Ghanaian patients of all ages treated with 3-day course of artesunate-amodiaquine

The capillary whole blood concentrations by day of follow up are shown in Fig. 2a and b, with the univariate analysis of pharmacokinetic parameters shown by age category in Table 4. The median area under the concentration–time curve to infinity (AUC_0-∞_) was 1318 (IQR 583–3,549) ng h/mL overall. Infants had a higher median (IQR) AUC_0-∞_ of 4,201 (2,773–17,909) ng h/mL, when compared to 1994 (642–5,199) ng h/mL in children aged 1–4 years and 875 (400–1434) ng h/mL in those aged ≥ 5 years (p < 0.001). Overall, the median maximum amodiaquine concentration (C_max_) was 18.8 (IQR 9.8–50.7) ng/mL, which was reached (T_max_) in a median of 2 (IQR 1–3) days. The median observed Cmax also decreased with age, from 49.6 (21.7–107.0) ng/mL in infants, to 27.7 (14.7–70.0) ng/mL in children aged 1–4 years and 11.6 (6.7–18.8) ng/mL in older children and adults (p < 0.001). Median apparent clearance was 25.9 (IQR 8.6–56.0) L/kg/h overall, which increased with age, p < 0.001. The effects of age on amodiaquine AUC_0-∞_ and on C_max_ remained statistically significant when the two study sites were analysed separately, and the effect of age on apparent clearance was significant among the 198 patients enrolled in the Navrongo site, but not the smaller sample of 25 patients enrolled in the Kintampo site (Additional file 2). Median apparent volume of distribution was 1,195 (IQR 416–2735) L/kg, which also increased with age, p = 0.008. The terminal elimination half-life of amodiaquine was 47.4 (32.4–72.8) hours and similar across age groups (Table 4).

**Table 4:** Pharmacokinetic parameters of amodiaquine and desethylamodiaquine (median, IQR) following artesunate-amodiaquine fixed-dose combination treatment of Ghanaian patients with uncomplicated *P. falciparum* malaria, by age category

Parameter	Age category (years)				p-value
	All ages	< 1	1–4	≥ 5	
	n = 223	n = 9	n = 125	n = 89	
Amodiaquine					
AUC_0-∞_ (ng h /ml)	1318 (583–3549)	4201 (2773–17,909)	1994 (642–5199)	875 (400–1,434)	< 0.001
C_max_ (ng/ml)	18.8 (9.8–50.7)	49.6 (21.7–107.0)	27.7 (14.7–70.0)	11.6 (6.7–18.8)	< 0.001
T_max_ (days)	2 (1–3)	1 (1–2)	2 (1–3)	2 (1–3)	0.71
Cl/F (L kg^−1^ h^−1^)	25.9 (8.6–56.0)	6.7 (1.4–11.2)	15.7 (7.0–47.1)	39.6 (18.7–76.9)	< 0.001
Vd/F (L kg^−1^)	1,195 (416–2,735)	325 (98–1141)	859 (359–2,613)	1,840 (861–3,656)	0.008
t_½_ (hours)	47.4 (32.4–72.8)	54.1 (24.6–148.7)	48.8 (32.6–87.9)	41.7 (32.4–56.1)	0.24

	n = 227	n = 9	n = 127	n = 91	
Desethylamodiaquine					
AUC_0-∞_ (ng h /ml)	116,100 (70,325–203,110)	163,020 (94,262–255,562)	124,508 (79,736–225,582)	105,596 (56,986–159,382)	0.057
C_max_ (ng/ml)	565 (349–827)	365 (266–665)	604 (377–937)	528 (260–720)	0.046
T_max_ (days)	3 (2–3)	2 (2–3)	3 (2–3)	3 (2–3)	0.33
Cl/F(L kg^−1^ h^−1^)	0.26 (0.16–0.46)	0.19 (0.11–0.31)	0.24 (0.16–0.41)	0.31 (0.16–0.56)	0.071
Vd/F (L kg^−1^)	67.5 (43.0–126.8)	57.0 (40.1–100.5)	62.3 (42.9–124.6)	72.9 (45.3–150.2)	0.49
t _½_ (hours)	196.4 (152.8–276.1)	237.8 (204.6–280.3)	187.6 (155.1–276.1)	200.1 (137.5–268.6)	0.60

IQR Interquartile range, AUC_0-∞_ Area under the concentration versus time curve from time zero to infinity, ng.h /ml nanogram hour per milliliter, C_max_ = Observed maximum concentration, ng/ml nanogram per milliliter, T_max_ time to maximum concentration, CL/f apparent clearance, Vd/f apparent volume of distribution, t_½_ = elimination half-life

After adjusting for pre-defined covariates (Table 5), there was a higher amodiaquine AUC_0-∞_ [Geometric Mean Ratio (GMR) 5.42 (95% CI 1.20–24.57), p = 0.029] and a trend towards a higher C_max_ [GMR 2.69 (95% CI 0.83–8.73), p = 0.099] in infants compared to those aged 5 years and older. There was a 4% increase in AUC_0-∞_, [GMR 1.04 (95% CI 1.01, 1.06), p = 0.001], and a 3% increase in C_max_ [GMR 1.03 (95% CI 1.01, 1.05), p = 0.001] for each 1 mg/kg increase in total dose administered. Fever at enrolment was associated with a significant increase in AUC_0-∞_ [GMR 1.44 (95% CI 1.01–2.04), p = 0.045], and C_max_ [GMR 1.57 (95% CI 1.18–2.09), p = 0.002] with a lower apparent clearance [GMR 0.69 (95% CI 0.49–0.99), p = 0.042]. There was an 84% lower AUC_0-∞_ [GMR 0.16 (95% CI 0.09–0.27), p < 0.001] and an 82% lower C_max_ [GMR 0.18 (95% CI 0.12–0.28), p < 0.001] in patients from Navrongo compared to patients from Kintampo. This is consistent with the almost sevenfold increase in apparent clearance [GMR 6.82 (95% CI 4.0–11.63), p < 0.001] and fourfold increase in the apparent volume of distribution [GMR 4.30 (95% CI 2.20–8.39), p < 0.001] in patients from Navrongo compared to Kintampo. Given that the elimination half-life was similar between the study sites, the differences observed by site could reflect differences in bioavailability.

**Table 5:** Multivariate analysis of log-transformed Amodiaquine and Desethylamodiaquine pharmacokinetic parameters following artesunate-amodiaquine fixed-dose combination treatment of Ghanaian patients with uncomplicated *P. falciparum* malaria (geometric Mean Ratios (GMR) and 95% Confidence Intervals (CI))

	GMR	95% CI	p-value	GMR	95% CI	p-value
Area under the Curve (AUC_0-∞)_	Amodiaquine (n = 196)			Desethylamodiaquine (n = 210)		
Age category: < 1 versus ≥ 5 years	*5.42*	*1.20 to 24.57*	*0.029*	1.12	0.38 to 3.29	0.84
Age category: 1–4 versus ≥ 5 years	1.85	0.60 to 5.71	0.28	0.81	0.36 to 1.79	0.60
Baseline Fever (Temperature: ≥ 37.5 °C)	*1.44*	*1.01 to 2.04*	*0.045*	1.13	0.87 to 1.47	0.37
Underweight-for-age: WAZ < − 2.0 vs WAZ ≥ − 2.0	0.82	0.45 to 1.48	0.50	1.15	0.72 to 1.84	0.55
Underweight-for-age: NA vs WAZ ≥ − 2.0	0.82	0.26 to 2.57	0.74	0.68	0.31 to 1.53	0.35
Dose (mg/kg)	*1.04*	*1.01 to 1.06*	*0.001*	*1.02*	*1.00 to 1.04*	*0.041*
Sex: Female (vs. male)	0.74	0.54 to 1.01	0.058	0.87	0.68 to 1.12	0.28
Parasite density < 100,000 (vs ≥ 100,000/ µL)	0.91	0.58 to 1.42	0.67	1.13	0.81 to 1.58	0.46
Anaemia: Hb < 8.0 g/dL	1.44	0.87 to 2.36	0.15	1.20	0.81 to 1.78	0.35
Site: Navrongo (vs Kintampo)	*0.16*	*0.09 to 0.27*	< *0.001*	1.12	0.73 to 1.71	0.61
Prior AQ use: Day 0 DEAQ > 20 ng/mL				1.23	0.93 to 1.63	0.14

Maximum concentration (Cmax)	Amodiaquine (n = 217)			Desethylamodiaquine (n = 211)		
Age category: < 1 versus ≥ 5 years	2.69	0.83 to 8.73	0.099	0.73	0.26 to 2.00	0.54
Age category: 1–4 versus ≥ 5 years	2.30	0.93 to 5.65	0.071	0.86	0.40 to 1.85	0.69
Baseline Fever (Temperature: ≥ 37.5 °C)	*1.57*	*1.18 to 2.09*	*0.002*	1.17	0.91 to 1.51	0.22
Underweight-for-age: WAZ < -2.0 vs WAZ ≥ − 2.0	0.63	0.38 to 1.05	0.075	1.08	0.69 to 1.70	0.72
Underweight-for-age: NA vs WAZ ≥ − 2.0	0.95	0.38 to 2.36	0.90	0.69	0.32 to 1.51	0.36
Dose (mg/kg)	*1.03*	*1.01 to 1.05*	*0.001*	1.01	1.00 to 1.03	0.14
Sex: Female (vs. male)	0.80	0.62 to 1.05	0.105	0.85	0.67 to 1.07	0.17
Parasite density < 100,000 (vs ≥ 100,000/µL)	0.77	0.53 to 1.13	0.18	1.09	0.79 to 1.50	0.62
Anaemia: Hb < 8.0 g/dL	*1.72*	*1.12 to 2.64*	*0.013*	1.23	0.85 to 1.79	0.27
Site: Navrongo (vs Kintampo)	*0.18*	*0.12 to 0.28*	< *0.001*	1.36	0.90 to 2.05	0.14
Prior AQ use: Day 0 DEAQ > 20 ng/mL				1.18	0.90 to 1.54	0.23

Apparent Clearance (Cl/f)	Amodiaquine (n = 196)			Desethylamodiaquine (n = 210)		
Age category: < 1 versus ≥ 5 years	*0.20*	*0.04 to 0.89*	*0.035*	0.95	0.32 to 2.81	0.92
Age category: 1–4 versus ≥ 5 years	0.55	0.18 to 1.71	0.30	1.27	0.57 to 2.84	0.56
Baseline Fever (Temperature: ≥ 37.5 °C)	*0.69*	*0.49 to 0.99*	*0.042*	0.88	0.67 to 1.15	0.34
Underweight-for-age: WAZ < -2.0 vs WAZ ≥ − 2.0	1.23	0.67 to 2.23	0.50	0.87	0.54 to 1.39	0.56
Underweight-for-age: NA vs WAZ ≥ − 2.0	1.22	0.39 to 3.84	0.73	1.47	0.65 to 3.33	0.35
Dose (mg/kg)	1.00	0.98 to 1.02	0.79	1.01	1.00 to 1.03	0.093
Sex: Female (vs. male)	1.34	0.98 to 1.85	0.070	1.13	0.88 to 1.45	0.34
Parasite density < 100,000 (vs ≥ 100,000/µL)	1.10	0.7 to 1.71	0.69	0.88	0.63 to 1.24	0.46
Anaemia: Hb < 8.0 g/dL	0.70	0.42 to 1.16	0.16	0.83	0.56 to 1.24	0.37
Site: Navrongo (vs Kintampo)	*6.82*	*4.00 to 11.63*	< *0.001*	0.97	0.63 to 1.50	0.91
Prior AQ use: Day 0 DEAQ > 20 ng/mL				0.81	0.61 to 1.07	0.13

Apparent Volume of distribution (Vd/f)	Amodiaquine (n = 152)			Desethylamodiaquine (n = 209)		
Age category: < 1 versus ≥ 5 years	0.62	0.07 to 5.39	0.66	0.74	0.19 to 2.82	0.65
Age category: 1–4 versus ≥ 5 years	0.67	0.11 to 4.21	0.67	0.84	0.32 to 2.19	0.72
Baseline Fever (Temperature: ≥ 37.5 °C)	0.67	0.42 to 1.09	0.10	0.71	0.50 to 1.00	0.050
Underweight-for-age: WAZ < − 2.0 vs WAZ ≥ − 2.0	1.44	0.7 to 2.99	0.32	0.91	0.50 to 1.65	0.76
Underweight-for-age: NA vs WAZ ≥ − 2.0	1.16	0.18 to 7.26	0.88	0.95	0.36 to 2.51	0.91
Dose (mg/kg)	1.01	0.98 to 1.04	0.42	*1.02*	*1.00 to 1.05*	*0.044*
Sex: Female (vs. male)	0.93	0.61 to 1.42	0.74	1.23	0.90 to 1.7	0.20
Parasite density < 100,000 (vs ≥ 100,000/µL)	0.78	0.43 to 1.4	0.41	1.19	0.76 to 1.85	0.45
Anaemia: Hb < 8.0 g/dL	0.54	0.28 to 1.05	0.070	0.75	0.45 to 1.25	0.27
Site: Navrongo (vs Kintampo)	*4.30*	*2.20 to 8.39*	< *0.001*	0.76	0.44 to 1.31	0.32
Prior AQ use: Day 0 DEAQ > 20 ng/mL				0.86	0.59 to 1.24	0.41

Elimination half-life (t½)	Amodiaquine (n = 152)			Desethylamodiaquine (n = 208)		
Age category: < 1 versus ≥ 5 years	1.82	0.59 to 5.6	0.29	1.05	0.49 to 2.21	0.91
Age category: 1–4 versus ≥ 5 years	1.78	0.75 to 4.18	0.19	0.90	0.52 to 1.53	0.69
Baseline Fever (Temperature: ≥ 37.5 °C)	0.95	0.72 to 1.24	0.70	0.85	0.70 to 1.03	0.103
Underweight-for-age: WAZ < − 2.0 vs WAZ ≥ − 2.0	0.98	0.65 to 1.48	0.92	0.95	0.68 to 1.32	0.74
Underweight-for-age: NA vs WAZ ≥ − 2.0	1.39	0.59 to 3.29	0.45	0.84	0.49 to 1.45	0.52
Dose (mg/kg)	1.01	0.99 to 1.02	0.54	1.01	1.00 to 1.02	0.18
Sex: Female (vs. male)	*0.74*	*0.58 to 0.94*	*0.013*	1.08	0.90 to 1.29	0.41
Parasite density < 100,000 (vs ≥ 100,000/µL)	0.84	0.61 to 1.17	0.31	*1.41*	*1.09 to 1.81*	*0.008*
Anaemia: Hb < 8.0 g/dL	0.94	0.64 to 1.36	0.73	0.91	0.69 to 1.21	0.52
Site: Navrongo (vs Kintampo)	0.85	0.58 to 1.24	0.39	0.86	0.63 to 1.16	0.31
Prior AQ use: Day 0 DEAQ > 20 ng/mL				1.08	0.88 to 1.32	0.48

WAZ Weight for Age z-score, NA Not applicable (WAZ score only calculated for children ≤ 5 years), AQ amodiaquine, DEAQ desethylamodiaquine,

### Desethylamodiaquine

**Fig. 3:**
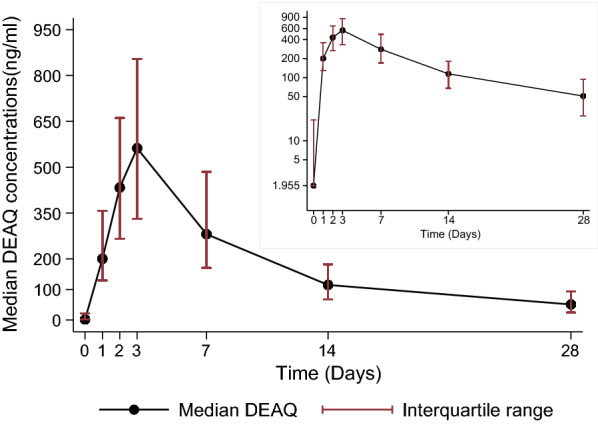
A plot of the median capillary whole blood desethylamodiaquine (DEAQ) concentrations versus time profile overlaid with log-transformed y-axis plot of the median (interquartile range) capillary whole blood desethylamodiaquine concentrations versus time in Ghanaian patients of all ages treated with 3-day course of artesunate-amodiaquine

**Fig. 4:**
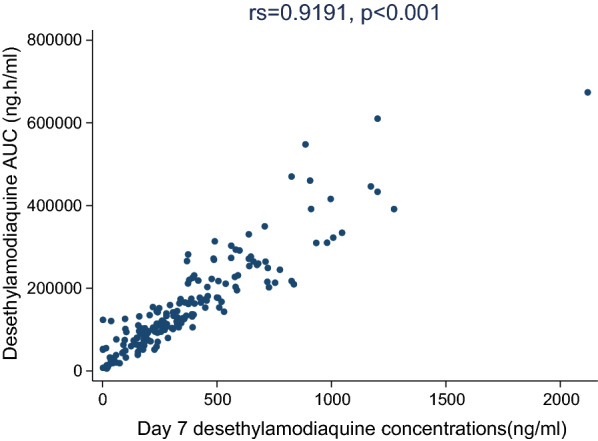
A scatter plot showing the correlation between the area under the capillary whole blood concentration–time curve of desethylamodiaquine (AUC0-∞) and the day 7 desethylamodiaquine (DEAQ) concentrations

The capillary whole blood concentrations of desethylamodiaquine by day of follow up are displayed in Fig. 3a, b and univariate analysis of pharmacokinetic parameters by age category presented in Table 4. Desethylamodiaquine concentrations were quantifiable in all patients throughout the 28-day follow up period. As expected, the peak concentrations and AUC_0-∞_ of desethylamodiaquine were much larger than those for amodiaquine. The desethylamodiaquine terminal elimination half-life of 196.4 h was four-fold longer than that for amodiaquine. Maximum desethylamodiaquine concentrations increased with age (p = 0.046), with a trend towards AUC_0-∞_ (p = 0.057) decreasing with age and apparent clearance increasing with age (p = 0.071). There was a strong linear correlation between the day 7 desethylamodiaquine concentrations and the AUC_0-∞_; r_s_ = 0.881, p < 0.001 (Fig. 4).

After adjusting for all other predefined covariates (Table 5), there was no association between desethylamodiaquine exposure and age or study site, in contrast with amodiaquine exposure. A 1 mg/kg increase in the total amodiaquine dose was associated with a 2% increase in the AUC_0-∞_[GMR 1.02 (95% CI 1.00, 1.04), p = 0.041], which is substantial given the bodyweight adjusted total dose range between 23 and 45 mg/kg. Although high baseline parasitaemia was associated with a 40% reduction in the elimination half-life compared with patients with parasite densities < 100,000/µL [GMR 1.40 (95% CI 1.09, 1.81), p = 0.008], no other pharmacokinetic parameters were affected by baseline parasite density (Table 5).

### Pharmacokinetic–pharmacodynamic relationship

**Fig. 5:**
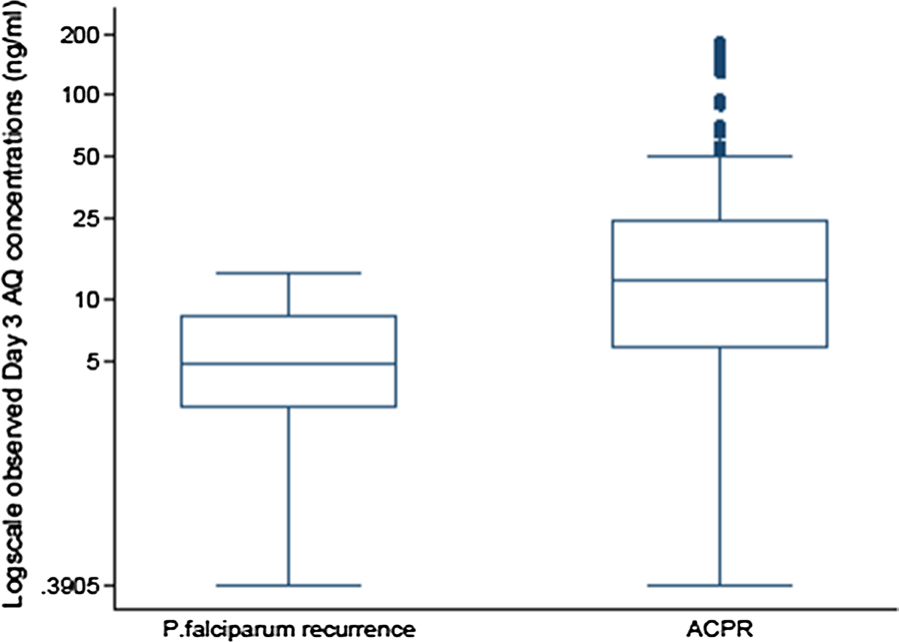
Box and whisker plots of log scale observed Day 3 amodiaquine concentrations by treatment response

Treatment efficacy was very high in this study with a PCR-adjusted adequate clinical and parasitological response rate of over 97%. The study was therefore underpowered to show any pharmacokinetic differences between patients who achieved an adequate clinical and parasitological response and those who failed treatment. Given the few treatment failures, treatment response categories were simplified to ACPR or treatment failure (i.e. any parasite recurrence). The median amodiaquine concentration on day 3 was significantly lower in patients with parasite recurrence than in those with an ACPR [4.8 (IQR 3.0–8.2) vs. 12.5 (IQR 5.9–24.2) ng/mL; p = 0.002, Fig. 5]. A 1 ng/mL increase in median day 3 amodiaquine concentration was associated with a 13% reduction in the risk of parasite recurrence, [HR = 0.8737 (95% CI 0.7793, 0.9795), p = 0.021]. The day 7 desethylamodiaquine concentrations, p = 0.767, as well as total desethylamodiaquine exposure, AUC_0-∞_, p = 0.363, were similar between patients who achieved ACPR and those with recurrent parasitaemia.

## Discussion

Amodiaquine is rapidly and extensively metabolized to desethylamodiaquine, its main active metabolite that has a much longer elimination half-life than amodiaquine and generally provides most of its anti-malarial effect. This study demonstrates that desethylamodiaquine exposure in whole blood is remarkably consistent across all age groups, which is reassuring since the highest *P. falciparum* malaria burden is carried by children under 5 years of age [2]. This is in contrast to findings for a number of other widely used anti-malarials including lumefantrine, piperaquine, and sulfadoxine-pyrimethamine that appear to have been systematically under-dosed in young children [37–39]. Equally reassuring is that desethylamodiaquine exposure is not reduced in underweight-for-age young children or those with a high parasitaemia, two of the most vulnerable target population groups.

The inclusion of infants with uncomplicated malaria in this study provides preliminary evidence that they may have greater exposure to amodiaquine than older children and adults. There was a significant decrease in total amodiaquine exposure and maximum concentration with age, even when study sites were analysed separately (Table 4 and Additional file 2). After adjusting for covariates, there was a higher amodiaquine total exposure, with a trend towards a higher maximum concentration and a slower apparent clearance in the 9 infants included in the pharmacokinetic analysis (Table 5). No safety concerns were identified in this study, despite the higher amodiaquine concentrations observed in infants relative to older children and adults. Consistent with the safety findings, the maximum amodiaquine concentrations observed in infants in this study were similar to whole blood concentrations reported after a single oral dose of 600 mg amodiaquine in 7 healthy adult males [29] and in plasma concentrations in Zambian and Nigerian malaria patients aged 7–55 years [17], and much lower than the plasma concentrations reported in Ugandan patients aged 1.5–8 years [40]. A larger prospective pharmacokinetic study including infants and young children with close monitoring of safety, including full blood counts and liver function tests, is needed to confirm the higher amodiaquine exposure in infants and understand any safety implications.

Age-dependent changes in body composition or maturational effects may contribute to the pharmacokinetic differences by age group [41], and amodiaquine and desethylamodiaquine clearance increase with age and bodyweight [42, 43]. Amodiaquine is primarily metabolised by Cytochrome P450 isoenzyme 2C8 (CYP2C8), and its expression increases with age [42–45]. However, classification tree analysis of CYP2C8 expression indicates two nodes for age stratification, namely 35 days and 11 years, with no indication of a difference in CYP2C8 expression between older infants (including all infants in this study whose ages range between 5.2 and 11.5 months) and children under 12 years of age [44].

Although there was a trend towards total desethylamodiaquine exposure and apparent clearance decreasing with age, its pharmacokinetic parameters did not differ significantly between age categories after adjusting for pre-defined covariates. The median capillary whole blood desethylamodiaquine AUC_0-∞_ in this study is similar to those obtained in whole blood spots from malaria patients aged 1–10 years in Papua New Guinea [18], but about 3-times higher than that previously reported in plasma samples in Ghanaian malaria patients aged 1–14 years [20]. Similarly, the whole blood amodiaquine AUC_0-∞_ in this study was substantially higher than the few published amodiaquine AUC_0-∞_ values reported, all of which were measured in plasma [22, 29, 46]. These differences may reflect matrix effects, with amodiaquine and desethylamodiaquine being concentrated in the white blood cells [24–26], and greater sensitivity of the assay developed in this study. Genotyping was not included in this study, so the contribution of slow metabolisers (CYP2C8*2) of amodiaquine could not be excluded, although previous studies suggest a prevalence between 16 and 18% in Ghana [20, 47, 48].

No safety concerns were identified in this study, with all adverse events mild to moderate and many consistent with malaria features. Although safety was assessed clinically at all scheduled and unscheduled study visits, it was not feasible to perform laboratory tests to monitor for neutropenia or hepatotoxicity, which have been associated with amodiaquine use particularly when taken as chemoprophylaxis or with interacting medicines [3, 49, 50]. The proportion of patients reporting adverse events in this study was lower than previously reported in children in the two study areas [8, 9]. One explanation may be that the co-blistered artesunate-amodiaquine tablets (used in the two other studies) have been associated with less accurate dosing, and higher doses may lead to more side effects [51, 52]. These differences may also reflect methods used in eliciting adverse events, with the inclusion of additional laboratory investigations (such as differential white blood cell counts and liver function tests) and different methods of questioning that have been shown to influence safety data [53].

In 2005, Ghana replaced chloroquine with the artemisinin-based combination treatment, artesunate-amodiaquine, given as loose tablets or co-blister packs for the treatment of uncomplicated malaria [4]. This study provides reassurance that the efficacy of the fixed dose combination of artesunate-amodiaquine remained high 7 to 8 years after its deployment in Ghana, with genotype-corrected ACPR rates by day 28 above 97% confirmed across all age categories and by both intention-to-treat and per protocol analysis, with only one patient (0.3%) still parasitaemic on microscopy on day 3. This may in part reflect the improved efficacy of the fixed dose combination. In a large pooled individual patient data meta-analysis of 9106 patients treated with artesunate-amodiaquine, the day 28 genotype-corrected ACPR rate of the fixed-dose combination was superior to loose tablet combinations [52]. However, there is the need for therapeutic efficacy studies to be repeated regularly and with optimal follow up [30, 54], in order to assess whether the efficacy of artesunate-amodiaquine has been sustained, particularly in the light of sporadic reports of indigenous artemisinin-resistant *P. falciparum* in Africa [55–57].

All patients in this study had quantifiable concentrations of desethylamodiaquine on day 28, showing that longer follow up would be needed to detect all treatment failures, as recrudescent parasitaemia may be suppressed by these quantifiable drug concentrations [24, 30, 54]. Earlier reports suggest that the formation of desethylamodiaquine from amodiaquine is rapid with very little of the parent drug, amodiaquine, being detectable in plasma beyond the third day post-dose [18, 29, 58]. However, similar to Ntale et al*.* [41], amodiaquine concentrations in this study were still quantifiable later in a substantial proportion (35.8%) of patients on day 7 and in 13% of patients on day 28, possibly reflecting the greater amodiaquine exposure in the infants and young children included, the greater sensitivity of the assay used and possibly also the relatively high prevalence of CYP2C8*2 in Ghana [20, 47, 48, 59].

Assessments of amodiaquine/desethylamodiaquine concentrations should be included in these therapeutic efficacy studies wherever possible, at least on day 7 and before starting treatment [60]. In holo-endemic areas like Ghana, repeated use of anti-malarials is common and often undisclosed [61]. This is reflected by over 20% of enrolled patients in this study having quantifiable amodiaquine concentrations prior to dosing despite excluding patients who reported recent anti-malarial treatment.

In sub-Saharan Africa, malaria and malnutrition often co-exist and are important public health conditions. It is estimated that 1 in 3 children under 5 years of age in sub-Saharan Africa suffer from malnutrition [62]. Malnutrition has been observed to alter the pharmacokinetic properties of chloroquine and quinine [3]. A pooled individual patient data analysis showed that Day 7 lumefantrine concentrations were lower and reinfection rates higher in underweight young children than adequately nourished children and adults; similar findings were seen in children with severe acute malnutrition when compared with well-nourished children [63, 64]. In this study however, the 28 (14.7%) underweight-for-age children aged ≤ 5 years did not have significant changes in amodiaquine or desethylamodiaquine pharmacokinetic parameters. Similarly, amodiaquine and desethylamodiaquine exposure was not altered in the 47 patients with a high parasitaemia at enrolment, despite their shorter elimination half-life. This provides reassuring evidence of the accuracy of the currently recommended dosing in these important vulnerable populations.

Study site appeared to be an important factor affecting the pharmacokinetic parameters of amodiaquine, despite adjustments for other predefined covariates. However, the pharmacokinetic properties of the active metabolite responsible for most anti-malarial activity, desethylamodiaquine were similar between sites. While the exact reason for the disparities observed by site of sample collection is not known, these differences may be consistent with higher amodiaquine bioavailability in Kintampo than Navrongo. Although it is possible that more patients Kintampo had non-protocol high-fat intake before dosing, this alone would not fully explain the apparent site effects. Compared with the fasted state in healthy adult volunteers, a high fat meal given before dosing had relatively modest effects, delaying absorption slightly and increasing both Cmax (by 22% for amodiaquine and 21% for desethylamodiaquine) and AUC (by 59% for amodiaquine and 13% for desethylamodiaquine) [65]. It has been suggested that ethnicity and concomitant medication use [59, 66–68] are among other factors that may account for unexplained site effects. Concomitant medication use reported in both study sites was similar, with paracetamol and iron/multivitamin supplements most commonly reported in both sites. Ethnically, however, the two study sites are distinct. The main indigenous groups in Kintampo are the Bono and the Mo, while the Kassena and the Nankana are the main two distinct ethno-linguistic groups in the Navrongo area. Ethnic differences could be explained by potential differences in prevalence of CYP2C8*2 but no data is available on the pharmacogenetic profiles of these ethnic groups.

A simple liquid–liquid extraction method coupled with LC–MS/MS detection that uses a small volume of whole blood (20 µL) was developed and fully validated in this study for the simultaneous determination of amodiaquine and its active metabolite, desethylamodiaquine. The method achieved lower limits of detection relative to the small sample volume, with good sensitivity and reproducibility. Small sample volumes may be necessary for field studies to ease sample collection, particularly from infants and small children. This method can readily be used for pharmacokinetic studies and therapeutic drug monitoring in patients and at-risk groups, including children to whom amodiaquine based combinations are given for the treatment or prevention of falciparum malaria.

## Conclusion

This large study of the pharmacokinetic properties of amodiaquine when used with artesunate for the treatment of uncomplicated malaria provides reassuring evidence of high cure rates with desethylamodiaquine exposure remarkably consistent across all age groups, including in underweight-for-age children and those with hyperparasitaemia. The inclusion of infants with uncomplicated malaria in this study provides preliminary evidence that they may have greater exposure to amodiaquine than older children and adults. Although no safety concerns were identified in this study, there is the potential for more adverse events in infants given their higher amodiaquine exposure and observed maximum concentrations (Cmax), particularly with increasing deployment of seasonal malaria chemoprophylaxis (SMC) in the Sahel region where 81 million courses of SP-amodiaquine were delivered in 2018 [69]. In addition to the need for heightened pharmacovigilance in infants treated with amodiaquine, a larger pharmacokinetic study with close monitoring of safety, including full blood counts and liver function tests, is needed to confirm the higher amodiaquine exposure in infants, understand any safety implications and assess whether dose optimization in this vulnerable, understudied population is needed.

## Supplementary Information

**Additional file 1.** Liquid chromatography tandem mass spectrometry assay methods.

**Additional file 2. **Pharmacokinetic parameters (median, IQR) of amodiaquine and desethylamodiaquine following artesunate-amodiaquine fixed-dose combination treatment of Ghanaian patients with uncomplicated *P. falciparum* malaria, by study site and age category.

## Data Availability

The datasets generated and/or analysed during the current study are available in the WorldWide Antimalarial Resistance Network (WWARN) repository [http://www.wwarn.org/].
