# Correction to: Pharmacokinetic profile of amodiaquine and its active metabolite desethylamodiaquine in Ghanaian patients with uncomplicated falciparum malaria

**DOI:** 10.1186/s12936-021-03651-z

**Published:** 2021-03-19

**Authors:** Thomas A. Anyorigiya, Sandra Castel, Katya Mauf, Frank Atuguba, Bernhards Ogutu, Abraham Oduro, David Dosoo, Kwaku-Poku Asante, Seth Owusu-Agyei, Alexander Dodoo, Abraham Hodgson, Fred Binka, Lesley J. Workman, Elizabeth N. Allen, Paolo Denti, Lubbe Wiesner, Karen I. Barnes

**Affiliations:** 1 Division of Clinical Pharmacology, Department of Medicine, University of Cape Town, Cape Town, South Africa; 2 UCT/MRC Collaborating Centre for Optimising Antimalarial Therapy (CCOAT), University of Cape Town, Cape Town, South Africa; 3 Navrongo Health Research Centre, Navrongo, Ghana; 4 Department of Statistical Sciences, University of Cape Town, Cape Town, South Africa; 5 Dodowa Health Research Centre, Dodowa, Ghana; 6 Centre for Clinical Research, Kenya Medical Research Institute, Nairobi, Kenya; 7 Kintampo Health Research Centre, Kintampo, Ghana; 8 University for Health and Allied Sciences, Ho, Volta Region Ghana; 9 Ghana Standards Authority, Accra, Ghana; 10 Research and Development Division, Ghana Health Service, Accra, Ghana

## Correction to: Malar J (2021) 20:18 https://doi.org/10.1186/s12936-020–03553-6

Following the publication of the original article [1], the authors noticed an error in the **units for Tmax** in the first of the two tables in **Additional file 2**, “Pharmacokinetic parameters (median, IQR) of amodiaquine and desethylamodiaquine following artesunate-amodiaquine fixed-dose combination treatment of Ghanaian patients with uncomplicated *P. falciparum* malaria, by study site and age category”.

In the table under sub-heading, **Navrongo Health Research Centre**:

i. The units of measure of Tmax (hours) for Amodiaquine should read **Tmax (days)**
ii. The units of measure of Tmax (hours) for Desethylamodiaquine should read **Tmax (days)**
iii. The units of t½ (days) for Desthylamodiaquine should read **t½ (hours)**

The file has been corrected in the original article and the correct version of the file can be found in this correction.

The authors apologize for any inconvenience caused.
