# Supplementary material for: Pharmacokinetic profile of amodiaquine and its active metabolite desethylamodiaquine in Ghanaian patients with uncomplicated falciparum malaria
Source: Malar J. 2021 Jan 6;20:18. doi: 10.1186/s12936-020-03553-6 (PMC7788723; doi:10.1186/s12936-020-03553-6)
Supplement: Supplementary file 1 — Additional file 1. Liquid chromatography tandem mass spectrometry assay methods. [file 12936_2020_3553_MOESM1_ESM.docx]

Additional file 1:
Liquid chromatography tandem mass spectrometry assay methods

The whole blood samples were thawed on ice and briefly vortexed. 200 µl of a Britton Robinson universal buffer (0.1 M, pH 8), containing both deuterated internal standards (amodiaquine-d10 and desethylamodiaquine-d5) at 1 ng/ml each, was added to each polypropylene tube into which 20 µl of the whole blood sample was aliquoted and 600 µl ethyl acetate added as the organic solvent. The samples were vortexed for 1 minute and centrifuged for 5 minutes at 16,000 rcf (relative centrifugal force). The organic phase (500 µL) was transferred to clean polypropylene tubes and evaporated under nitrogen at room temperature for 20 minutes. Reconstitution solution (100 µL), which consisted of acetonitrile and water with 0.1% formic acid (50:50, v/v), was added to the dry samples. The samples were vortexed for 30 seconds, transferred to a 96 well polypropylene plate, and 2 µL were injected onto the HPLC column.

The extraction procedure was followed by isocratic liquid chromatography on a Phenomenex Luna PFP (5 µm, 50 mm x 2.0 mm) analytical column with acetonitrile and water containing 0.1% formic acid (86:14, v/v) as the mobile phase, at a flow rate of 500 µL/min. An AB Sciex API 4000 mass spectrometer at unit resolution in the multiple reaction monitoring mode was used to monitor the transition of the protonated precursor ions m/z 356.2, m/z 366.2, m/z 328.0, and m/z 333.2 to the product ions m/z 283.1, m/z 283.1, m/z 283.1, and m/z 283.1 for amodiaquine, amodiaquine-d10, desethylamodiaquine and desethylamodiaquine-d5, respectively.

Electro Spray Ionisation was used for ion production and performed in the positive ion mode with nitrogen as the nebulizing, turbo spray and curtain gas, with the optimum values set at 65, 60 and 30 psi, respectively. The heated nebulizer temperature was set at 500 °C and the ionspray voltage set at 3500 V. The pause time was set at 5 msec, the dwell time at 150 msec, and the collision gas (N2) was set to medium to minimize background noise. The AB Sciex API 4000 mass spectrometer was operated at unit resolution in the multiple reaction monitoring (MRM) mode. The retention times for amodiaquine, desethylamodiaquine and their internal standards were ~ 1.3 and ~ 1.4 minutes, respectively. The total run time was 4.0 minutes. (See Supplementary tables S1.1-1.3).

The calibration curve fitted quadratic (weighted by 1/concentration2) regressions over the range 0.781 to 100 ng/mL for amodiaquine and 3.91 to 2000 ng/mL for desethylamodiaquine. The combined accuracy (%Nom) statistics of the quality controls of amodiaquine and desethylamodiaquine during validation were between 93.9% and 108.3%. The precision (total-assay coefficients of variation; CV %) during sample analysis were less than 6 % at high, medium and low (75, 37.5 and 2.34 ng/ml, respectively) QC levels for amodiaquine, and were less than 8 % at high, medium and low (1500, 750 and 5.86 ng/ml, respectively) levels for desethylamodiaquine (See Supplementary Material Tables S1.4 – 1.7).

Benchtop stability (on ice) was assessed at low and high standards for 6 hours (maximum anticipated time that future study samples will be left thawed until extracted). These samples were analysed against a valid calibration curve. The precision and % Difference for the analyte and metabolite are reported to be within 15%, indicating stability for at least 6 hours on ice. No additional storage stability assessments were performed, as the analyte and metabolite are reported to be stable in human whole blood under the storage conditions used. ^[[1]](#footnote-1),^^[[2]](#footnote-2)^

AB Sciex API 4000 mass spectrometer Settings

*Table S1.1: Electro Spray Ionisation Settings*

| **Nebuliser gas (Gas 1) (arbitrary unit)** | 65 |
| --- | --- |
| **Turbo gas (Gas 2) (arbitrary unit)** | 60 |
| **CUR (curtain gas) (arbitrary unit)** | 30 |
| **CAD (collision gas) (arbitrary unit)** | 6 |
| **TEM (Source Temperature) (°C)** | 500 |
| **IS (Ion Spray Voltage) (V)** | 3500 |

*Table S1.2: MS/MS Settings*

|  | **AQ** | **AQ-ISTD** | **DAQ** | **DAQ-ISTD** |
| --- | --- | --- | --- | --- |
| Protonated molecular ion mass (m/z) [M+H]^+^ | 356.20 | 366.25 | 328.09 | 333.28 |
| Product ion mass (m/z)   Quantifier | 283.10 | 283.10 | 283.10 | 283.10 |
| Product ion mass (m/z)   Qualifier | 255.10 | 219.00 | 219.10 | 219.10 |
| Dwell time (ms) | 100 | 100 | 100 | 100 |
| Declustering potential (V) | 66 | 76 | 56 | 66 |
| Entrance potential (V) | 10 | 10 | 10 | 10 |
| Collision energy (eV) | 25 | 27 | 23 | 25 |
| Collision cell exit potential (V) | 24 | 20 | 20 | 24 |

*Table S1.3: Scan Description*

| Scan Type | MRM |
| --- | --- |
| Polarity | Positive |
| Pause Time (ms) | 5 |

Statistics for assay validation:

Table S1.4: Accuracy and Precision Estimation for Amodiaquine QCs

| **Validation** | **Sample ID** | **QC LLOQ-1** | **QC LLOQ-2** | **QC Low** | **QC Med** | **QC High** |
| --- | --- | --- | --- | --- | --- | --- |
| **Batch** | **Nominal Conc.** | **0.781** | **1.56** | **2.34** | **37.5** | **75** |
|  |  | **(ng/ml)** | **(ng/ml)** | **(ng/ml)** | **(ng/ml)** | **(ng/ml)** |
|  | **Replicates** | **Observed Conc.** | | | | |
|  | **n** | 18 | 18 | 18 | 18 | 18 |
|  | **Average** | 0.808 | 1.47 | 2.30 | 40.6 | 80.8 |
|  | **STDEV** | 0.0378 | 0.0523 | 0.136 | 1.81 | 3.08 |
|  | **% CV** | 4.8 | 3.4 | 5.8 | 4.8 | 4.1 |
|  | **% Accuracy** | 103.5 | 93.9 | 98.2 | 108.3 | 107.7 |

Table S1.5: Overall Summary of Calibration Standard Accuracy and Precision: Validation 1-3 for Amodiaquine

| **Validation** | **Sample ID** | **ULOQ STD 1** | **STD 2** | **STD 3** | **STD 4** | **STD 5** | **STD 6** | **STD 7** | **LLOQ STD 8** |
| --- | --- | --- | --- | --- | --- | --- | --- | --- | --- |
| **Batch** | **Nominal Conc.** | **100** | **50.0** | **25.0** | **12.5** | **6.25** | **3.13** | **1.56** | **0.781** |
|  |  | **(ng/ml)** | **(ng/ml)** | **(ng/ml)** | **(ng/ml)** | **(ng/ml)** | **(ng/ml)** | **(ng/ml)** | **(ng/ml)** |
|  | **Replicates** | **Observed Conc.** | | | | | | | |
|  | **Average** | 97.0 | 52.2 | 26.3 | 12.8 | 6.10 | 2.90 | 1.52 | 0.806 |
|  | **STDEV** | 1.59 | 1.76 | 0.627 | 0.223 | 0.163 | 0.0532 | 0.0366 | 0.0198 |
|  | **% CV** | 1.6 | 3.4 | 2.4 | 1.7 | 2.7 | 1.8 | 2.4 | 2.5 |
|  | **% Accuracy** | 97.0 | 104.4 | 105.3 | 102.5 | 97.7 | 92.5 | 97.3 | 103.2 |

Table S1.6: Accuracy and Precision Estimation for Desethylamodiaquine QCs

| **Validation** | **Sample ID** | **QC LLOQ** | **QC Low** | **QC Med** | **QC High** |
| --- | --- | --- | --- | --- | --- |
| **Batch** | **Nominal Conc.** | **3.91** | **5.86** | **750** | **1500** |
|  |  | **(ng/ml)** | **(ng/ml)** | **(ng/ml)** | **(ng/ml)** |
|  | **Replicates** | **Observed Conc.** | | | |
|  | **n** | 18 | 18 | 18 | 18 |
|  | **Average** | 3.97 | 5.63 | 794 | 1547 |
|  | **STDEV** | 0.194 | 0.260 | 27.7 | 48.1 |
|  | **% CV** | 5.0 | 4.4 | 3.7 | 3.2 |
|  | **% Accuracy** | 101.5 | 96.1 | 106 | 103.1 |

Table S1.7: Overall Summary of Calibration Standard Accuracy and Precision: Desethylamodiaquine

| **Validation** | **Sample ID** | **ULOQ STD 1** | **STD 2** | **STD 3** | **STD 4** | | **STD 5** | **STD 6** | **STD 7** | | **STD8** | **STD 9** | **LLOQ STD 10** |
| --- | --- | --- | --- | --- | --- | --- | --- | --- | --- | --- | --- | --- | --- |
| **Batch** | **Nominal Conc.** | **2000** | **1000** | **500** | **250** | | **125** | **62.5** | **31.3** | | **15.6** | **7.81** | **3.91** |
|  |  | **(ng/ml)** | **(ng/ml)** | **(ng/ml)** | **(ng/ml)** | | **(ng/ml)** | **(ng/ml)** | **(ng/ml)** | | **(ng/ml)** | **(ng/ml)** | **(ng/ml)** |
|  | **Replicates** |  | | | |  | | | | **Observed Conc.** | | | |
|  | n | 6 | 6 | 6 | 6 | | 6 | 6 | 6 | | 6 | 6 | 6 |
|  | Average | 1910 | 1060 | 536 | 266 | | 126 | 60.3 | 29.9 | | 14.5 | 7.32 | 4.13 |
|  | STDEV | 51.8 | 29.0 | 21.7 | 14.1 | | 4.00 | 1.55 | 0.750 | | 0.175 | 0.356 | 0.154 |
|  | % CV | 2.7 | 2.7 | 4.0 | 5.3 | | 3.2 | 2.6 | 2.5 | | 1.2 | 4.9 | 3.7 |
|  | % Accuracy | 95.5 | 106.0 | 107.1 | 106.3 | | 100.8 | 96.5 | 95.4 | | 92.7 | 93.7 | 105.6 |
|  |  |  |  |  |  |  |  |  |  |  |  |  |  |

Representative Chromatograms

Figure S1.1: Representative Chromatogram of amodiaquine at the lower limit of concentration (0.781 ng/ml)


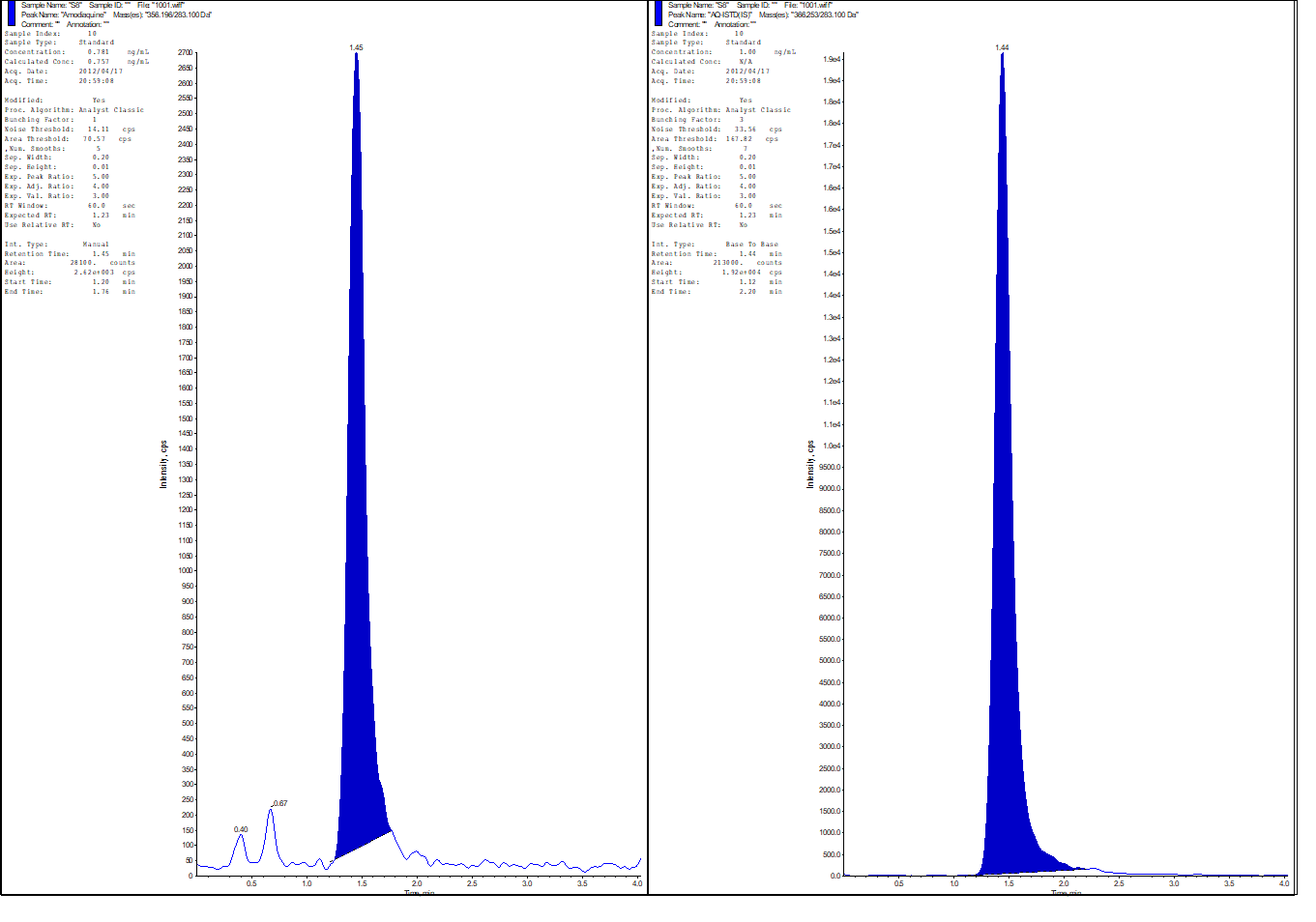


Figure S1.2: Representative Chromatogram of desethylamodiaquine at the lower limit of concentration (15.6 ng/ml)


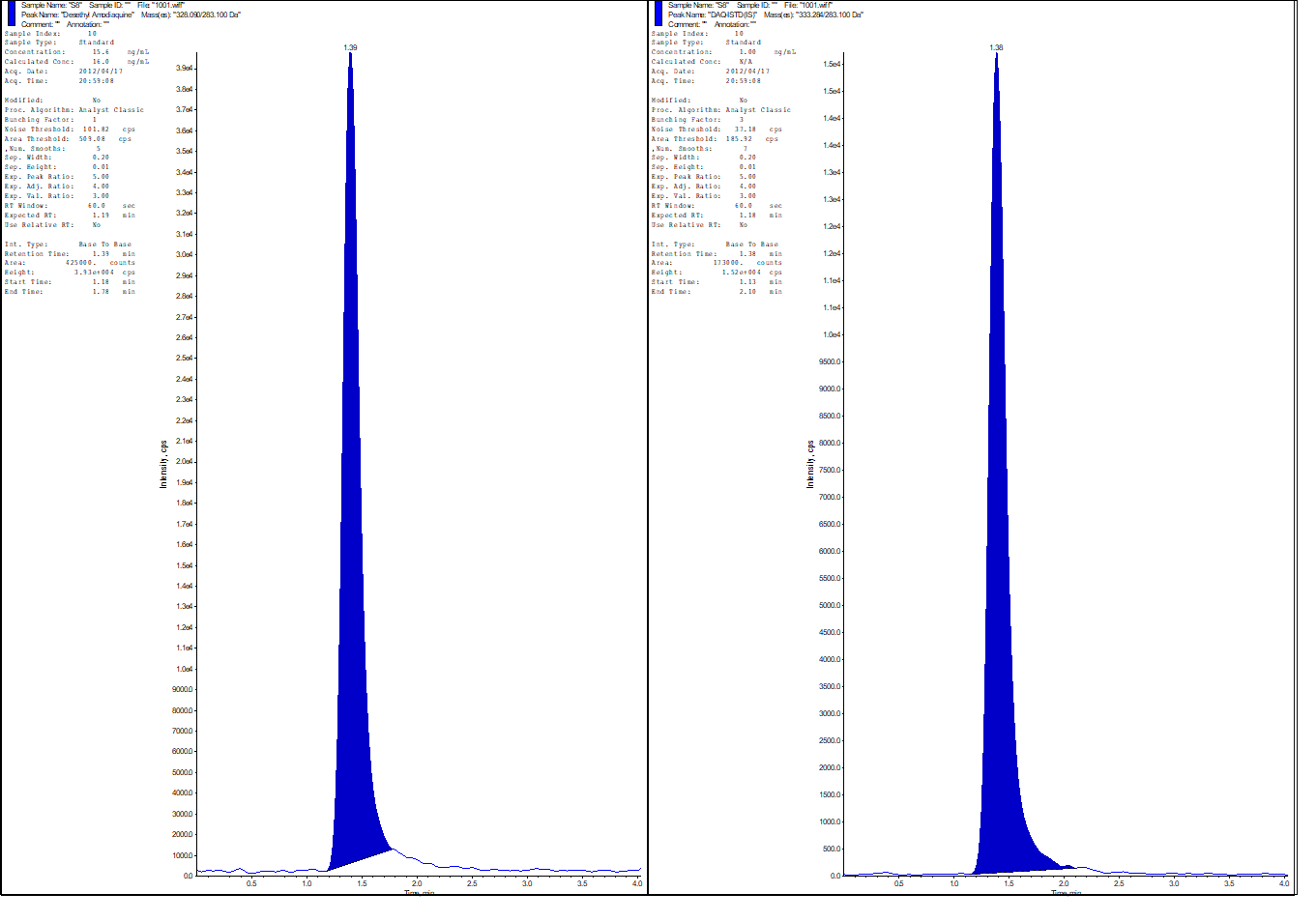


Figure S1.3: Representative Chromatogram of amodiaquine mid-level quality control sample (37.5 ng/ml)


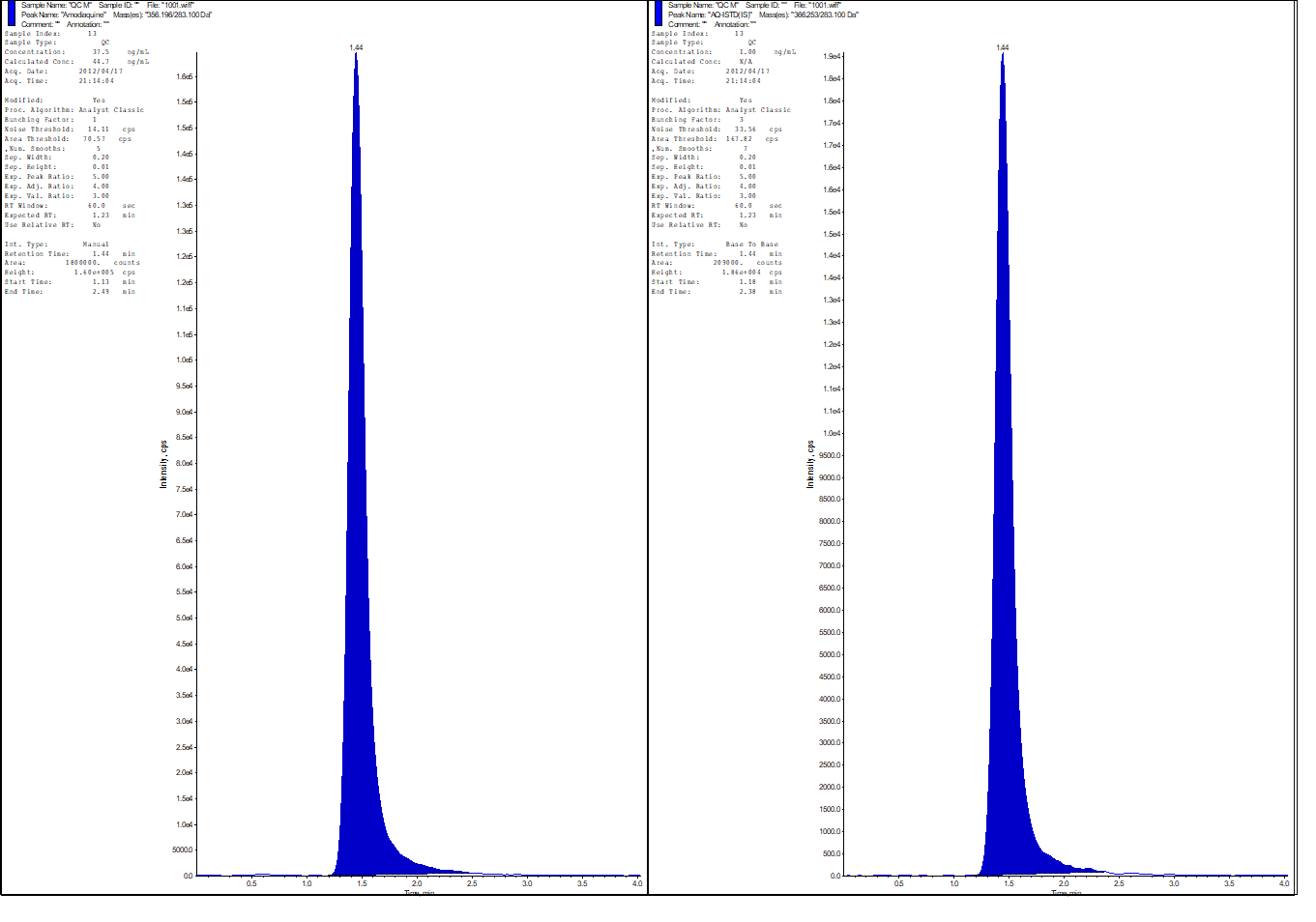


Figure S1.4: Representative Chromatogram for desethylamodiaquine mid-level quality control sample (750 ng/ml)


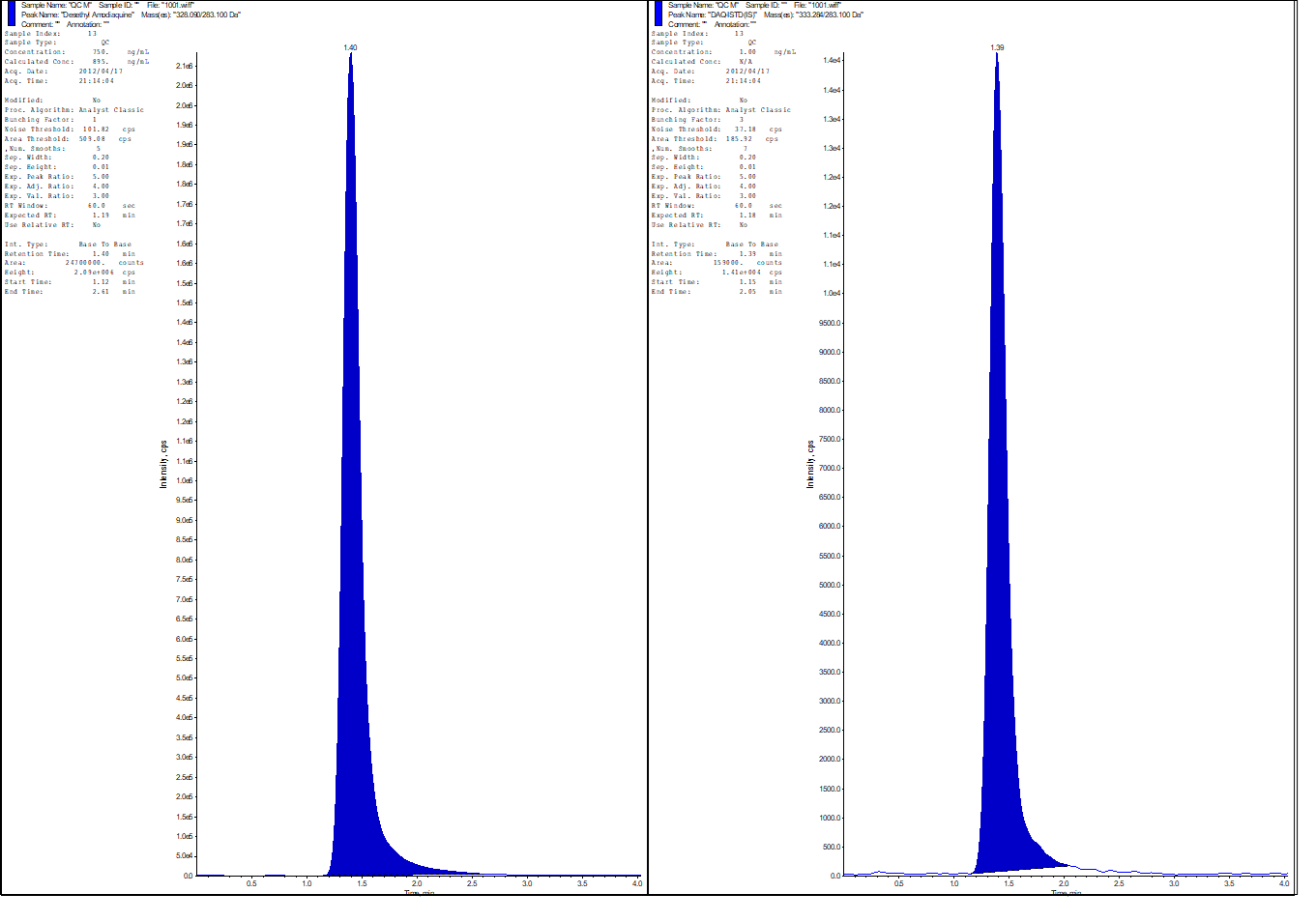


Figure S1.5: Representative Patient Chromatogram for amodiaquine


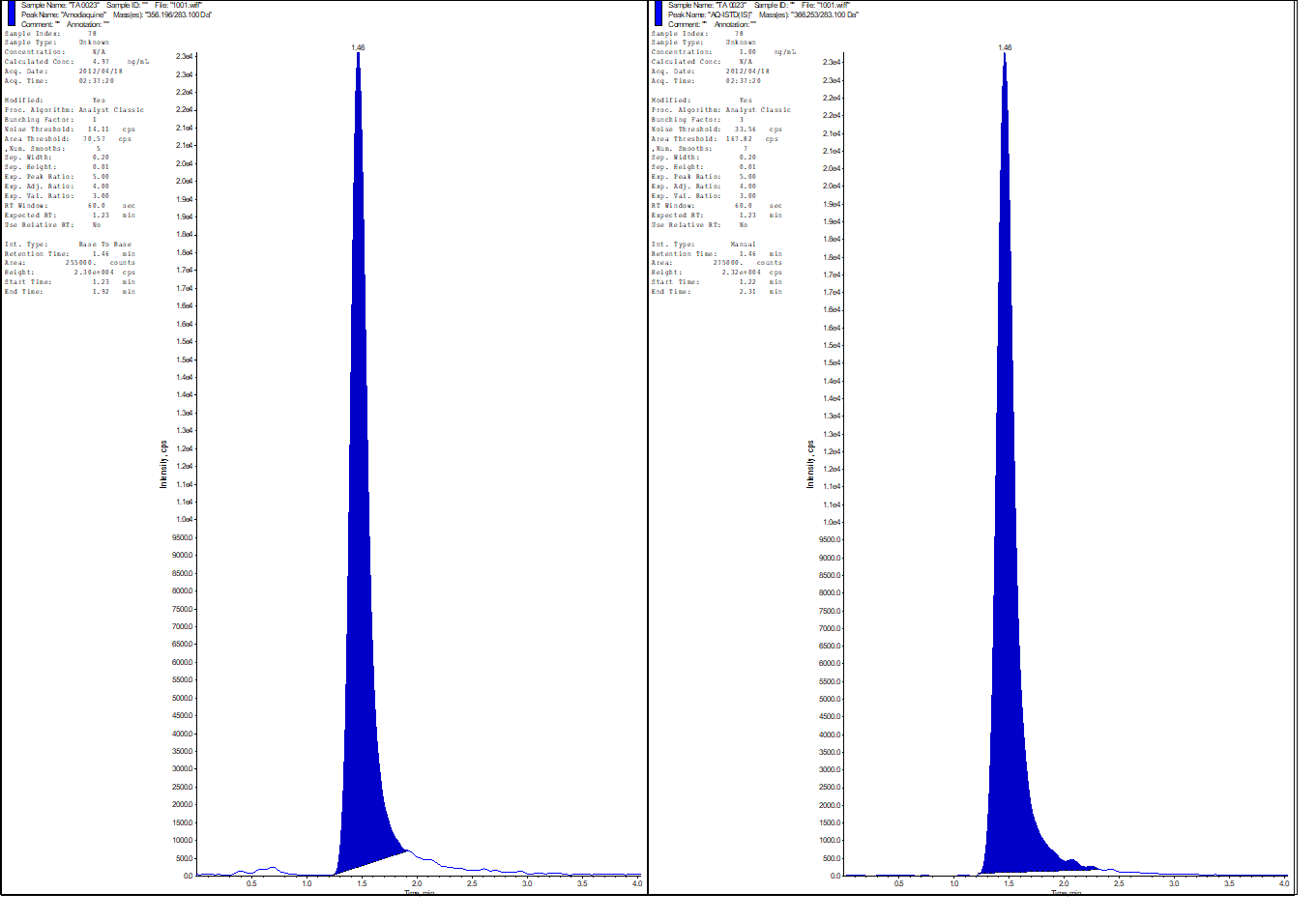


Figure S1.6: Representative Patient Chromatogram for desethylamodiaquine


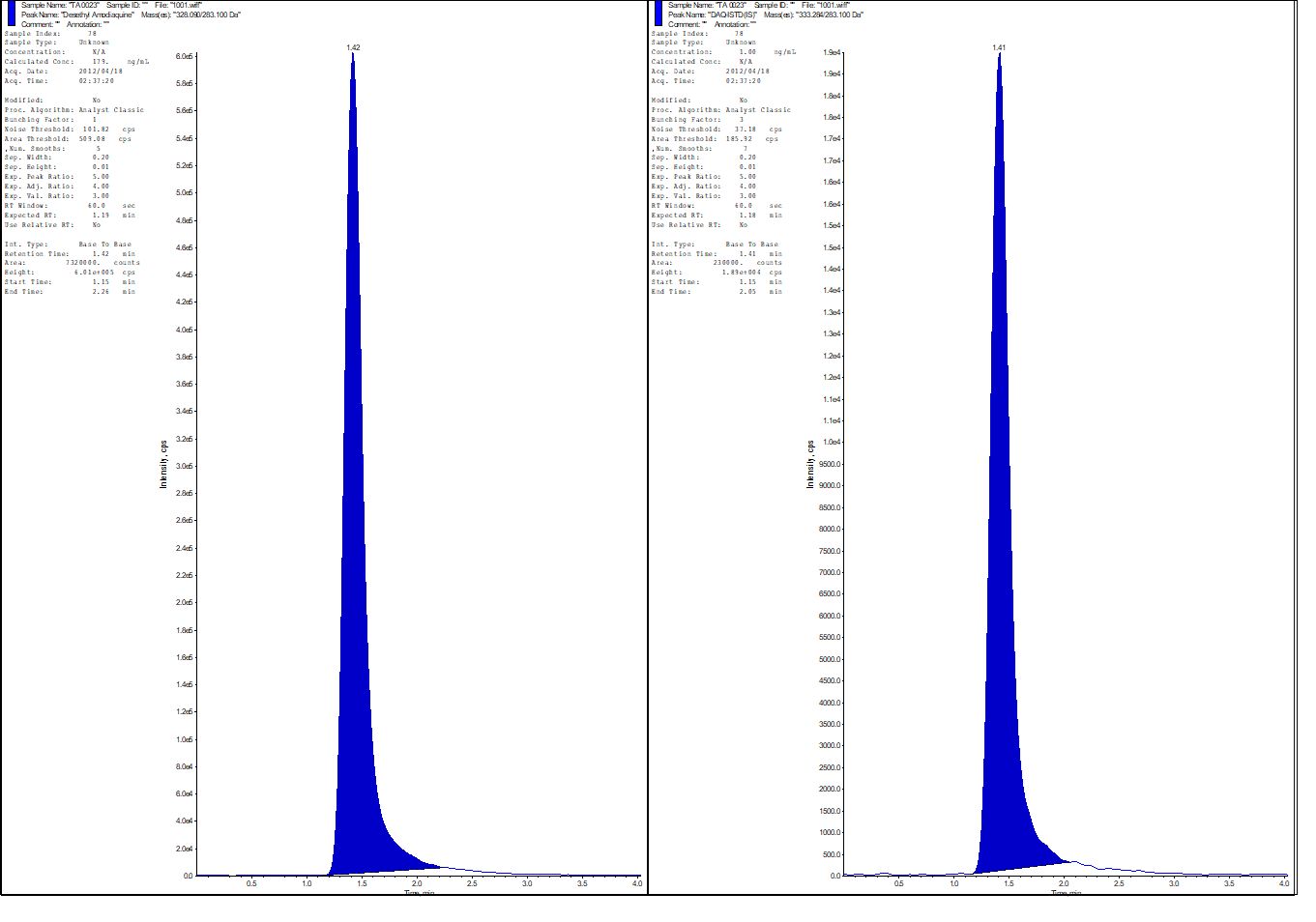


1. *Blessborn D, Neamin G, Bergqvist Y, Lindegårdh N. A new approach to evaluate stability of amodiaquine and its metabolite in blood and plasma. J Pharm Biomed Anal. 2006 Apr 11;41(1):207-12.* [↑](#footnote-ref-1)
2. *Rathod DM, Patel KR, Mistri HN, Jangid AG, Shrivastav PS, Sanyal M. Application of an LC-MS/MS method for reliable determination of amodiaquine, N-desethylamodiaquine, artesunate and dihydroartemisinin in human plasma for a bioequivalence study in healthy Indian subjects. J Pharm Biomed Anal. 2016 May 30;124:67-78.* [↑](#footnote-ref-2)
