# Supplementary material for: Pharmacokinetic profile of amodiaquine and its active metabolite desethylamodiaquine in Ghanaian patients with uncomplicated falciparum malaria
Source: Malar J. 2021 Jan 6;20:18. doi: 10.1186/s12936-020-03553-6 (PMC7788723; doi:10.1186/s12936-020-03553-6)
Supplement: Supplementary file 2 — Additional file 2. Pharmacokinetic parameters (median, IQR) of amodiaquine and desethylamodiaquine following artesunate-amodiaquine fixed-dose combination treatment of Ghanaian patients with uncomplicated P. falciparum malaria, by study site and age category. [file 12936_2020_3553_MOESM2_ESM.docx]

**Additional file 2:**

**Pharmacokinetic parameters (median, IQR) of amodiaquine and desethylamodiaquine following artesunate-amodiaquine fixed-dose combination treatment of Ghanaian patients with uncomplicated *P.* *falciparum* malaria, by study site and age category**

|  | **Navrongo Health Research Centre** | | | | |
| --- | --- | --- | --- | --- | --- |
|  | **All ages** | **< 1 year** | **1 to 4 years** | **≥ 5 years** | **P-value** |
| **Amodiaquine** | *n=198* | *n=6* | *n=112* | *n=80* |  |
| AUC_0-∞_ (ng•h /ml) | 1,090  (517 – 2,735) | 3,162  (2296 - 4201) | 1,556  (615 – 3,983) | 803  (369 – 1,396) | <0.001 |
| C_max_ (ng/ml) | 17.4  (9.0 - 37.2) | 29.5  (19.8 - 49.6) | 25.4  (12.7 - 51.7) | 10.2  (6.4 - 17.8) | <0.001 |
| T_max_ (hours) | 2  (1 - 3) | 1.5  (1 - 3) | 2  (1 – 3) | 2  (1 - 3) | 0.76 |
| Cl/F (L.kg^-1^.h^-1^) | 27.9  (12.2 - 61.9) | 9.7  (6.7 - 12.2) | 22.5  (8.4 - 48.1) | 41.3  (26.1 - 88.8) | <0.001 |
| Vd/F( L. kg^-1^) | 1,401.7  (544.6 – 3,572.5) | 769.7  (325.4 – 4,872.3) | 1,089.1  (460.8 – 2,685.2) | 2,001.1  (1,232.6 – 4,018.6) | 0.022 |
| t_½_ (hours) | 46.5  (32.1 - 67.2) | 42.5  (23.0 - 601.6) | 48.0  (32.4 - 78.1) | 43.5  (32.0 - 55.2) | 0.38 |
| **Desethylamodiaquine** | *n=202* | *n=6* | *n=114* | *n=82* |  |
| AUC_0-∞_ (ng•h /ml) | 116,426  (70,325 – 209,674) | 163,020  (96,190 – 209,674) | 125,919  (80,162 – 225,806) | 106,163.2  (56026.8 - 163486.2) | 0.065 |
| C_max_ (ng/ml) | 587  (365 - 891) | 423  (266 - 917) | 609  (391 – 1,022) | 539  (260 - 807) | 0.064 |
| T_max_ (hours) | 3  (2 - 3) | 3  (2 - 3) | 3  (2 - 3) | 3  (2 – 3) | 0.69 |
| Cl/F(L.kg^-1^.h^-1^) | 0.26  (0.16 - 0.48) | 0.19  (0.11 - 0.26) | 0.24  (0.16 - 0.41) | 0.32  (0.18 - 0.61) | 0.038 |
| Vd/F ( L. kg^-1^) | 67.0  (430 - 133.2) | 57.0  (51.5 - 90.3) | 60.8  (42.3 - 124.6) | 73.7  (45.8 - 154.2) | 0.31 |
| t_½_ (days) | 190.6  (140.1 - 277.8) | 237.8  (206.6 - 257.4) | 186.9  (152.8 - 276.1) | 191.2  (131.3 - 279.3) | 0.77 |

|  | **Kimtampo Health Research Centre** | | | | |
| --- | --- | --- | --- | --- | --- |
|  | **All ages** | **< 1 year** | **1 to 4 years** | **≥ 5 years** | **P-value** |
| **Amodiaquine** | *n=25* | *n=3* | *n=13* | *n=9* |  |
| AUC_0-∞_ (ng•h /ml) | 5,666  (2297 – 17,909) | 42,010  (17,909 – 66,111) | 7,523  (4,788 – 35,009) | 2,172  (1,313 – 4,554) | 0.016 |
| C_max_ (ng/ml) | 81.0  (50.7 – 220.0) | 196.0  (107.0 – 423.0) | 82.9  (73.5 – 329.0) | 32.5  (15.1 – 60.2) | 0.027 |
| T_max_ (days) | 1  (1 - 2) | 1  (1 - 2) | 1  (1 - 2) | 1  (1 – 1) | 0.89 |
| Cl/F (L.kg^-1^.h^-1^) | 4.48  (1.20 - 6.49) | 0.90  (0.38 - 1.41) | 3.38  (0.90 - 5.92) | 5.62  (4.01 - 16.21) | 0.072 |
| Vd/F( L. kg^-1^) | 274.6  (102.5 - 858.3) | 89.8  (82.1 - 97.5) | 183.3  (107.5 - 802.9) | 380.3  (326.7 – 1,183.5) | 0.12 |
| t_½_ (hours) | 51.1  (38.9 - 99.7) | 98.3  (47.8 - 148.7) | 64.1  (39.4 - 155.6) | 40.6  (34.2 - 81.5) | 0.37 |
| **Desethylamodiaquine** | *n=25* | *n=3* | *n=13* | *n=9* |  |
| AUC_0-∞_ (ng•h /ml) | 105,523  (70,058 – 162,154) | 164,948  (74,333 – 255,563) | 107,311  (73,474 – 173,784) | 103,656  (66,642 – 153,326) | 0.60 |
| C_max_ (ng/ml) | 442  (266 - 599) | 357  (224 - 665) | 442  (266 - 658) | 489  (297 - 565) | 0.88 |
| T_max_ (days) | 2  (2 - 3) | 2  (1 - 2) | 2  (2 - 3) | 3  (2 - 3) | 0.078 |
| Cl/F(L.kg^-1^.h^-1^) | 0.29  (0.13 - 0.38) | 0.22  (0.10 - 0.34) | 0.27  (0.16 - 0.67) | 0.30  (0.13 - 0.33) | 0.56 |
| Vd/F ( L. kg^-1^) | 80.6  (40.1 - 112.6) | 70.3  (40.1 - 100.5) | 89.2  (49.5 - 289.1) | 72.2  (39.7 - 86.1) | 0.57 |
| t_½_ (hours) | 211.1  (179.8 - 251) | 242.4  (204.6 - 280.3) | 200.9  (178.7 - 251.8) | 213.0  (196.4 – 236.0) | 0.77 |
